# Where in the world: Mapping medical student learning using the Social and Structural Determinants of Health Curriculum Assessment Tool (SSDH CAT)

**DOI:** 10.1080/10872981.2023.2178979

**Published:** 2023-03-12

**Authors:** Karen Sheehan, Tami R. Bartell, Ashti A. Doobay-Persaud, Mark D. Adler, Karen A. Mangold

**Affiliations:** a Departments of Pediatrics, Medical Education, and Preventive Medicine, Feinberg School of Medicine, Northwestern University; Ann & Robert H. Lurie Children’s Hospital of Chicago, Chicago, Illinois, USA; b Research Project Manager, Patrick M. Magoon Institute for Healthy Communities, Ann & Robert H. Lurie Children’s Hospital of Chicago, Chicago, Illinois, Chicago; c Departments of Medicine and Medical Education, Feinberg School of Medicine, Northwestern University; d Departments of Pediatrics and Medical Education, Feinberg School of Medicine, Northwestern University; Ann & Robert H. Lurie Children’s Hospital of Chicago, Chicago, Illinois, USA

**Keywords:** Social determinants of health, structural determinants of health, evaluation, assessment, undergraduate medical education, global health, curriculum

## Abstract

**Introduction:**

Addressing the Social and Structural Determinants of Health (SSDH) is a primary strategy for attaining health equity. Teaching and learning about SSDH has increased across medical schools throughout the world; however, the published literature describing these efforts continues to be limited and many unknowns persist including what should be taught and by whom, what teaching methods and settings should be used, and how medical learners should be assessed.

**Materials and Methods:**

Based on published studies, input from experts in the field, and elements from the framework developed by the National Academy of Medicine, we created a universal Social and Structural Determinants of Health Curriculum Assessment Tool (SSDH CAT) to assist medical educators to assess existing SSDH curricular content, ascertain critical gaps, and categorize educational methods, delivery, and assessment techniques and tools that could help inform curricular enhancements to advance the goal of training a health care workforce focused on taking action to achieve health equity. To test the usefulness of the tool, we applied the SSDH CAT to map SSDH-related curriculum at a US-based medical school.

**Results:**

By applying the SSDH CAT to our undergraduate medical school curriculum, we recognized that our SSDH curriculum relied too heavily on lectures, emphasized knowledge without sufficient skill building, and lacked objective assessment measures. As a result of our curricular review, we added more skill-based activities such as using evidence-based tools for screening patients for social needs, and created and implemented a universal, longitudinal, experiential community health curriculum.

**Discussion:**

We created a universal SSDH CAT and applied it to assess and improve our medical school’s SSDH curriculum. The SSDH CAT provides a starting point for other medical schools to assess their SSDH content as a strategy to improve teaching and learning about health equity, and to inspire students to act on the SSDH.

## Introduction

It is estimated that only 10–20% of a population’s health outcomes are influenced by medical care [1]. Rather, it is the social and economic conditions, collectively referred to as the social determinants of health (SDH) [2], that have been shown to have a much greater influence on the health of patients and communities worldwide [3,4]. In addition, the often unnamed structural determinants of health or the ‘cultural norms, policies, institutions, and practices that define the distribution and maldistribution of the SDH’ (Crear-Perry et al., 2021, p. 231) [5] are recognized to represent the root causes of health inequities leading to an unequal distribution of SDH and ill health across global communities and racial and ethnic groups.

Due to the large impact of social and structural determinants of health (SSDH) [4,6], medical education can and should engage students to understand not just what the SSDH are but how they perpetuate health inequities and how to act on them to achieve social change.

For decades, leading health organizations from around the world have called for action [7–10] toward building a health care workforce competent to address the SSDH; and numerous groups have responded [11–16]. To further this process, in 2016 The National Academies of Science, Engineering, and Medicine (NASEM) [17] shared a conceptual model [18] and provided a universal framework for educating health professionals in understanding SSDH, with a specific call for educators to ‘review, map, and align their educational and professional vision, mission, and standards’ to include the SSDH (pg. 10). However, while U.S.-based medical schools have started to implement teaching about the SSDH [19,20], as now required by the Liaison Committee on Medical Education (LCME) [21] and outlined by the Association of American Medical Colleges (AAMC) [22], the published literature describing this work has been limited [23] and has not definitively answered what should be taught, what teaching methods should be used, or how methods and learners should be assessed, leading to a lack of social accountability [24,25]. As noted by Sharma et al. (2018), ‘A key challenge in understanding how the SDH are taught in medical schools is locating the SDH in medical curriculum’ (p. 25) [26].

To help answer these questions, we aimed to create a universal SSDH Curricular Assessment Tool (SSDH CAT) to support medical school educators in identifying existing SSDH content, methods, and assessment techniques to inform curricular enhancements.

## Materials and methods

### Tool development

The SSDH CAT was informed by a scoping review of published studies on teaching medical students about the SDH [23] and a modified Delphi process [27] to identify the knowledge, skills and attitudes (KSA) that students at US medical schools should optimally attain to address the SSDH. KSA topics that received a score ≥4 (1 = low, 5 = high) from the Delphi panel and elements of the NASEM framework [17] were integrated into the tool (Supplemental Table 1). An overview of how to use the SSDH CAT is provided in the Appendix.

### Analysis

The authors iteratively discussed and refined the tool and multiple drafts were circulated among authors for review. To test the tool’s usefulness, we applied the SSDH CAT to map SSDH-related pre-clinical curriculum for the 2016–2017 academic year at Northwestern University Feinberg School of Medicine, a large, research intensive US medical school; the medical school trains ~160 students per year. We reviewed learning guides and teaching materials to document what was taught (i.e., effect on cardiovascular health) and how it was taught (i.e., small group or lecture). These findings were used to inform curricular enhancements in subsequent academic years through 2022. Analysis of implementation and outcome data is ongoing.

## Results

As a result of implementing the SSDH CAT over the past 5 years, we recognized that the curriculum was missing discussions about structural inequality, effects of community violence on mental health, and differences in life expectancy by neighborhood. We updated content that lacked context, i.e., describing disparities for different disease states without clearly discussing upstream factors (i.e., racism, oppression), and potential solutions. We also identified that our SSDH curriculum relied too heavily on lectures, emphasized knowledge without sufficient skill building, and lacked objective assessment measures. As a result, we added more skill-based activities, such as using evidence-based tools to screen for SSDH [28,29] and providing students access to NowPow [30], an electronic referral platform to identify community-based resources. A major result was the implementation of a universal, longitudinal 4-year community health project focused on developing skills (i.e., using data for planning and principles of partnership and collaboration) to support action to improve community health. A manuscript describing the outcomes of this project is in development.

## Discussion

We developed the SSDH CAT to inform SSDH curriculum in undergraduate medical school education. At our institution, the SSDH CAT helped identify gaps and highlighted the need for additional skills-based SSDH activities and expanded experiential learning opportunities.

There will likely be local and geographic variation in curricular content. For example, based on data from a community health needs assessment [31] and community collaborations we know that medical students in Chicago, IL, USA, should learn about how racial segregation and ‘red lining’, intergenerational poverty, lack of access to quality mental health services, and easy access to firearms have contributed to high rates of community violence, as a way to attain structural competency [32].

Global, national and local strategies for educating medical students about the SSDH must also consider the historical and sociopolitical context of each individual country and its interrelationships [33–35]. There is strong support for the integration of global health competencies in medical education [36], as global and local structures drive health inequities [37]. While a focus on SSDH education shifts the gaze away from the role of clinical care, there is still need for awareness of elements of clinical medicine, in the US and other countries, that perpetuate health inequities and undermine optimal care [38,39].

Research to inform the SSDH CAT was limited to published studies from North America and US-based experts. While the same forms of structural violence [40] that create health inequities in low- and middle-income countries are also present in high-income countries including the US [41], there has historically been a greater body of research linking those structures to health disparities in disease in the Global South [42,43]. Future studies should include a more diverse range of voices and approaches [14,44].

Completing the SSDH CAT can provide a starting point for medical schools trying to increase KSA in addressing SSDH. Medical educators will be prompted to consider not only what is important to teach about the SSDH, but how to teach, structure, and assess it, and how to guide students to take action to meaningfully address issues of health equity. While this will differ across locations, within and across countries, and more work will be needed to refine content and evaluate effects on trainees and real-world impact, applying the SSDH CAT can help in this process.

## Supplementary Material

Supplemental Material

## Appendix

### Guide to Using the Social and Structural Determinants of Health Curriculum Assessment Tool (SSDH CAT)

#### Description of the SSDH CAT

The SSDH CAT is a self-administered tool that can be used by medical schools to examine components of their SSDH curriculum. Completion of SSDH CAT provides educators with more than a checklist of what SSDH are taught, but an overall curriculum blueprint of ongoing and new curriculum that identifies where and how specific aspects of SSDH are taught and assessed. In this way, the SSDH CAT provides a first step to self-assess strengths and weaknesses as they relate to teaching medical students about SSDH to gain structural competency [37]. As defined by the LCME: ‘Structural competency … refers to the capacity for health professionals to recognize and respond to the role that social, economic, and political structural factors play in patient and community health (Element 7.6).’ [26]

A SSDH curriculum that is effectively integrated throughout all years of medical school requires identification and assessment of all components of the SSDH domains [22].

The SSDH CAT was organized into domains as identified by consensus from an expert multidisciplinary panel using a Delphi process [32] and after reflection on available tools [22,28].

There are three sections to the SSDH CAT:

- Section I. Knowledge, Skills, Attitudes (KSA) for the identified domains: Forces and Systems; Social and Neighborhood Context and Conditions; Education Access and Quality; Economic Stability; Neighborhood and Built Environment; and Health Care Access and Quality
- Section II. Logistics including timing, duration, learner characteristics, educational methods, training locations, student levels of assessment, and objective levels of assessment
- Section III. School and Student Facilitators and Barriers

#### What the SSDH CAT Does NOT Do

While use of the SSDH CAT provides an overview of where SSDH-related curricula are offered within the curriculum, it is not intended to be prescriptive or allow for in-depth analysis of teaching strategies or learning outcomes but to serve as a framework. The SSDH CAT does not make recommendations for the optimal number of hours to be devoted to each domain or the entire curriculum. The SSDH CAT does not address the ‘informal curriculum’ that may influence student learning or achievement of structural competency.

#### Instructions for Completing the SSDH CAT

This guide is based on the experience of the authors who have used the tool in their own medical school setting.

- For Section I, educators should write in the names of courses, lectures, workshops, blocks, or clerkships based on their own curricular structure to identify what KSA are being addressed in each domain. Educators can also note the years that the content is addressed. If content is not included, responses should be ‘not addressed’. Responses in this section provide a quick snapshot of what and when SSDH are covered in the curriculum.
- For Section II, educators should identify the timing, duration, learner characteristics, educational methods, training locations, student levels of assessment, and objective levels of assessment for each element identified in Section I. There may be some overlap. For example, in Section I, whereas educational methods were listed, here educators may choose to explore more about which, how much and when each method is used, etc.
- For Section III, educators should identify the school and student facilitators and barriers listed that apply to their programs.

While comprehensive, the SSDH CAT is not an exhaustive list of SSDH; additional items can be added on the horizontal axis to any section and should reflect local variation in curricular content. Educators also can add more columns to collect additional information, i.e., to provide examples to support and/or clarify responses, as needed. Schools should also look to develop a glossary of terms to help ensure consistency of terms used and information collected.

Individual responses should be collated and areas ‘not addressed’ should be identified. A facilitator can provide a summary of the results including where content is missing. Other patterns that may emerge include lack of assessment in different domains, content in a single domain through multiple courses and/or a single course/clerkship where the majority of content is covered, etc. Following completion and discussion of the results of the SSDH CAT, schools may choose to undertake a more detailed analysis of their curriculum. To enrich the SSDH CAT, schools may opt to conduct focus groups to add depth to their planning process.
