# Supplementary material for: Where in the world: Mapping medical student learning using the Social and Structural Determinants of Health Curriculum Assessment Tool (SSDH CAT)
Source: Med Educ Online. 2023 Mar 12;28(1):2178979. doi: 10.1080/10872981.2023.2178979 (PMC10013438; doi:10.1080/10872981.2023.2178979)
Supplement: Supplemental Material [file ZMEO_A_2178979_SM5545.pdf]

<table>
<tr><td colspan="2" rowspan="3">SOCIAL AND STRUCTURAL DETERMINANTS OF HEALTH CURRICULAR ASSESSMENT TOOL (SSDH CAT)

This is a self-administered tool to identify current content and strategies for teaching SSDH in undergraduate medical education (UME):
What are we teaching and why? How are we teaching it? When is it offered? Who are the learners? What is the outcome/impact on students, patients, communities, society?</td><td colspan="4">TIMING

Mark X if topic is addressed</td><td rowspan="3">EDUCATIONAL METHODS

Didactic, case-based, small group, peer teaching, service learning, etc. Educators can also note specific curriculum used, links to reading, etc.</td></tr>
<tr><td colspan="4">YEAR</td></tr>
<tr><td>1</td><td>2</td><td>3</td><td>4</td></tr>
<tr><td colspan="2">SECTION I. KNOWLEDGE OF SOCIAL AND STRUCTURAL INEQUITIES AND THEIR CAUSES</td><td></td><td></td><td></td><td></td><td></td></tr>
<tr><td rowspan="5"><u>DOMAIN</u>
Forces and Systems

Institutions
Ideologies
Inequities</td><td>Social: racism--segregation, redlining, bigotry, oppression, violence; sexism, classism, ageism, ableism; discrimination based on sexual preference, gender identity, religion, national origin, genetic information, pregnancy</td><td></td><td></td><td></td><td></td><td></td></tr>
<tr><td>Economic: income inequality; fiscal policy, taxation; poverty reducing policies; disinvestment in communities</td><td></td><td></td><td></td><td></td><td></td></tr>
<tr><td>Health care: historical assessment of medical system, current health care system, insurance, community health needs assessments and implementation plans, etc.</td><td></td><td></td><td></td><td></td><td></td></tr>
<tr><td>Legal: immigration policies; criminal justice and injustice: mass incarceration, school discipline policies</td><td></td><td></td><td></td><td></td><td></td></tr>
<tr><td>Other:</td><td></td><td></td><td></td><td></td><td></td></tr>
<tr><td colspan="7"></td></tr>
<tr><td rowspan="5"><u>DOMAIN</u>
Social and Neighborhood Context and Conditions

Education Access and Quality

Economic Stability</td><td>Assets: culture, neighborhood resources and generational history; social cohesion, social capital; civic participation</td><td></td><td></td><td></td><td></td><td></td></tr>
<tr><td>Housing: availability, affordability, quality (e.g., exposure to lead and other toxins, air conditioning and ventilation, overcrowding)</td><td></td><td></td><td></td><td></td><td></td></tr>
<tr><td>Educational attainment: Access to high quality educational opportunities, availability of early childhood education; literacy, health literacy; child opportunity index (COI)</td><td></td><td></td><td></td><td></td><td></td></tr>
<tr><td>Economic stability: employment opportunities; equitable pay, schedules, workload; job security, access to benefits including paid leave and health insurance; food security; poverty and intergenerational poverty</td><td></td><td></td><td></td><td></td><td></td></tr>
<tr><td>Violence; victimization of and exposure to community violence; safety</td><td></td><td></td><td></td><td></td><td></td></tr>
</table>

	Data and surveillance: differences in life expectancy by neighborhood; results of Community Health Needs Assessments					
	Other:					
<u>DOMAIN</u><br>**Neighborhood and Built Environment**	Access to healthy food: availability and proximity of grocery stores, fresh fruit and vegetables; gardens					
	Access to physical features of built environment: neighborhood walkability and outlets for physical activity, green space, sidewalks, trails, parks, playgrounds, gyms, etc.					
	Access to health care: proximity to hospitals, mental health clinics, pharmacies, state of the art technology; community-based services for trauma-informed care, addiction support, harm reduction; safety net					
	Availability of affordable housing; quality, density, foreclosures					
	Availability, accessibility, affordability of high-speed internet service, telemedicine access					
	Availability and accessibility of public transportation					
	Environmental exposures: presence of lead in water, housing; air quality, proximity to highways, factories; exposure to elements of climate change					
	Other:					
<u>DOMAIN</u><br>**Health Care Access and Quality**<br><br>Systems<br>Providers<br>Settings	Access to timely, high quality and appropriate healthcare, guideline-concordant care and evidence-based practice; availability of primary and specialty care including mental health and dental care services<br>Quality of care: patient-provider interactions, appointment availability and duration, referral completion; over/under care, use of high-tech equipment; recruitment to clinical research studies and trials; bioethics<br>Health care costs					

	Geographic factors: distance, transportation to care settings; rural centers					
	Insurance coverage and costs: policies, eligibility, reimbursements rates; out of pocket costs, medical debt/bankruptcy; health literacy of patients/communities					
	Health center policies: charity care; implementation of Community Health Needs Assessments; onsite police presence; interactions with ICE,					
	DEI: cultural humility, explicit and implicit bias; providers of concordant race and ethnicity; language proficiency of providers, availability of interpretation services					
	Other:					
SECTION I. **SKILLS** FOR ACTING ON HEALTH INEQUITIES						
	Work effectively with community service providers such as community health workers					
	Work effectively as a member of a multidisciplinary team					
	Screen patients for assets and social needs					
	Apply advocacy skills					
	Solidarity with patients, communities					
	Develop patient care strategies based on SSDH					
	Access community resources					
	Leverage partnerships to Improve health					
	Use data effectively for care planning					
	Implement community engagement strategies					
	Identify multi-sector community resources					
	Work with medical-legal partnerships and/or legal aid clinics to address health harming legal needs					
	Understand and interpret CHNA findings and implementation strategies					
	Challenge status quo, develop solutions					
	Other:					
SECTION I. **ATTITUDES** FOR ACTING ON INEQUITIES AND HEALTH DISPARITIES						
	Appreciation of the impact of bias on medical decision making; role of race-based algorithms used in care					

	Extent of the impact of medical care alone; care occurring inside the health system is small component of what impacts a patient's overall health and well-being					
	Patient health and well-being in the context of family, culture, community, and society					
	Recognition of the social capital of physicians and health care providers in interactions with patients, communities, media, and policymakers to improve health					
	Appreciation of the interaction between individual and population health					
	Appreciation of the impact of collective advocacy by professional organizations to improve health					
	Need for a community-informed perspective					
	Role of physician in health system and in society—power, and privilege, benefits from status quo					
	Role as advocates and allies					
	Importance of health care system's role in identifying and prioritizing community health needs; knowledge of efforts to identify and prioritize community health needs					
	Other:					
**SECTION II. LOGISTICS**						
**Timing** *Our SDH curriculum is:*	Continuous over entire training	Yes No				
	Integrated into the rest of the curriculum	Yes No				
	Hybrid approach of integration and independence	Yes No				
	Pre-clinical	Yes No				
	Clinical	Yes No				
**Duration**	Short-term (≤ 6 weeks)	Yes No				
	Intermediate (6 weeks-1 year)	Yes No				
	Longitudinal (> 1 year)	Yes No				
**Learner Characteristics**	Elective	Yes No				
	Selective	Yes No				

Educational Methods	Didactic	More Less	
	Case-based	More Less	
	Small group	More Less	
	Peer teaching	More Less	
	Service learning	More Less	
	Critical reflection, critical consciousness	More Less	
	Community expert engagement, mentorship; community-based education/experience; contextualized experiential learning opportunities	More Less	
	Advocacy project		
	Multidisciplinary learning environment (nursing, social work, law, others)		
	Research		
	Theatre, film, literature; social media		
	Other:		
Training Locations	Campus	Yes No	
	Community sites: safety net, urban/rural sites	Yes No	
	Virtual	Yes No	
	Other:		
Student Levels of Assessment	Affective	Yes No	
	Self-reported changes in SDH Knowledge, Skills, Attitudes	Yes No	
	Objective Performance Assessment	Yes No	
	Reflection	Yes No	
	Other:		
Objective Levels of Assessment	Objective Clinical Skills Examination	Yes No	
	Patient feedback to student	Yes No	
	Community service provider feedback	Yes No	
	Measures of improved community health	Yes No	
	Other:		

SECTION III. SCHOOL AND STUDENT FACILIITATORS AND BARRIERS			
	Social responsibility; social accountability of medical school, medical system	Yes No	
	Student engagement in curriculum design, evaluation	Yes No	
	Community engagement in curriculum design, evaluation	Yes No	
	Community partnerships/collaborations	Yes No	
	Promotion and salary support for faculty, community leaders focused on equity/advocacy	Yes No	
	Faculty development – teaching/assessing SSDH education	Yes No	
	Faculty recruitment, retention – diversity, equity, inclusion	Yes No	
	Leadership support for equity curriculum	Yes No	
	Expanded roles for educators who are advocates, activists	Yes No	
	Admissions policies/procedures; DEI data and initiatives	Yes No	
	Metrics for how societal needs are being met by research, education and service	Yes No	
	Funding	Yes No	
	Other:		

*This tool is based on guidance from The National Academies of Science, Engineering, and Medicine (NASEM)'s framework for educating health professionals to address the social determinants of health,[8] and findings from a scoping review[16] to identify published studies about teaching the SSDH in UME and a Delphi process[17] to reach expert consensus on what should be taught about SSDH in UME.

<u>Instructions for Completing the SSDH-CAT</u>

- For Section I, educators should write in the names of courses, lectures, workshops, blocks, or clerkships based on their own curricular structure to identify what KSA are being addressed in each domain. Educators can also note the years that the content is addressed. If content is not included, responses should be 'not addressed'. Responses in this section provide a quick snapshot of what and when SSDH are covered in the curriculum.
- For Section II, educators should identify the timing, duration, learner characteristics, educational methods, training locations, student levels of assessment, and objective levels of assessment for each element identified in Section I. There may be some overlap. For example, in Section I, whereas educational methods were listed, here educators may choose to explore more about which, how much and when each method is used, etc.
- For Section III, educators should identify the school and student facilitators and barriers listed that apply to their programs.

While comprehensive, the SSDH-CAT is not an exhaustive list of SSDH; additional items can be added on the horizontal axis to any section and should reflect local variation in curricular content. Educators also can add more columns to collect additional information, i.e., examples to support and/or clarify responses, as needed. Schools should also look to develop a glossary of terms to help ensure consistency of terms used and information collected. Individual responses should be collated and areas 'not addressed' should be identified. A facilitator can provide a summary of the results including where content is missing. Other patterns that may emerge include lack of assessment in different domains, content in a single domain through multiple courses and/or a single course/clerkship where most content is covered, etc. Following completion and discussion of the results of the SSDH-CAT, schools may choose to undertake a more detailed analysis of their curriculum. To enrich the SSDH-CAT, schools may opt to conduct focus groups to add depth to their planning process.

For more information, please contact the SSDH-CAT creator: ksheehan@luriechildrens.org
